# Role of β-Catenin Activation Levels and Fluctuations in Controlling Cell Fate

**DOI:** 10.3390/genes10020176

**Published:** 2019-02-25

**Authors:** Elisa Pedone, Lucia Marucci

**Affiliations:** 1 Department of Engineering Mathematics, University of Bristol, Bristol, BS8 1UB, UK; Elisa.pedone@bristol.ac.uk; 2 School of Cellular and Molecular Medicine, University of Bristol, Bristol, BS8 1TD, UK; 3 BrisSynBio, Bristol, BS8 1TQ, UK

**Keywords:** canonical Wnt pathway, β-catenin, signaling dynamics, development, pluripotency, cancer

## Abstract

Cells have developed numerous adaptation mechanisms to external cues by controlling signaling-pathway activity, both qualitatively and quantitatively. The Wnt/β-catenin pathway is a highly conserved signaling pathway involved in many biological processes, including cell proliferation, differentiation, somatic cell reprogramming, development, and cancer. The activity of the Wnt/β-catenin pathway and the temporal dynamics of its effector β-catenin are tightly controlled by complex regulations. The latter encompass feedback loops within the pathway (e.g., a negative feedback loop involving Axin2, a β-catenin transcriptional target) and crosstalk interactions with other signaling pathways. Here, we provide a review shedding light on the coupling between Wnt/β-catenin activation levels and fluctuations across processes and cellular systems; in particular, we focus on development, in vitro pluripotency maintenance, and cancer. Possible mechanisms originating Wnt/β-catenin dynamic behaviors and consequently driving different cellular responses are also reviewed, and new avenues for future research are suggested.

## 1. Introduction

Wnt proteins are key mediators of cell specification and patterning in development, adult tissue homeostasis, and stemness [1]. Mutations of Wnt pathway components can cause a wide range of diseases, including congenital disorders [2,3,4,5,6,7,8,9,10,11,12,13] and cancer [10,14,15,16,17,18,19,20,21,22,23,24]. Wnts, conserved in all metazoan animals, can trigger the activation of two distinct signaling pathways, known as “canonical” and “noncanonical” [25]. The most studied Wnt pathway, and the focus of this review, is the canonical Wnt signaling; β-catenin, the pathway key effector and transcriptional coactivator, mediates canonical Wnt pathway functions. A detailed review about noncanonical Wnt signaling can be found in Reference [26].

β-catenin has a central role in directing diverse intracellular functions. It is involved in cell–cell adhesion through interaction with the E-cadherin cell-adhesion complex and the microtubule network [27,28,29,30,31,32,33,34], and can also trigger gene expression in complex with T-cell transcription factor/lymphocyte enhancer factor (TCF/LEF) family members [35,36,37,38,39,40,41,42,43,44].

The amount of β-catenin protein not complexed with E-cadherin is buffered by the destruction complex. The latter is a multiprotein complex; it consists of scaffolding proteins Axin, tumor suppressor APC, the serine–threonine kinases glycogen synthase kinase 3 (GSK3β), and the casein kinase 1 (CK1), and it is responsible for cytosolic β-catenin phosphorylation, ubiquitination, and degradation [45,46,47,48,49,50,51,52,53,54,55]. Upstream regulations of the destruction complex result in different accumulation of β-catenin in the cytosol.

**Figure 1:**
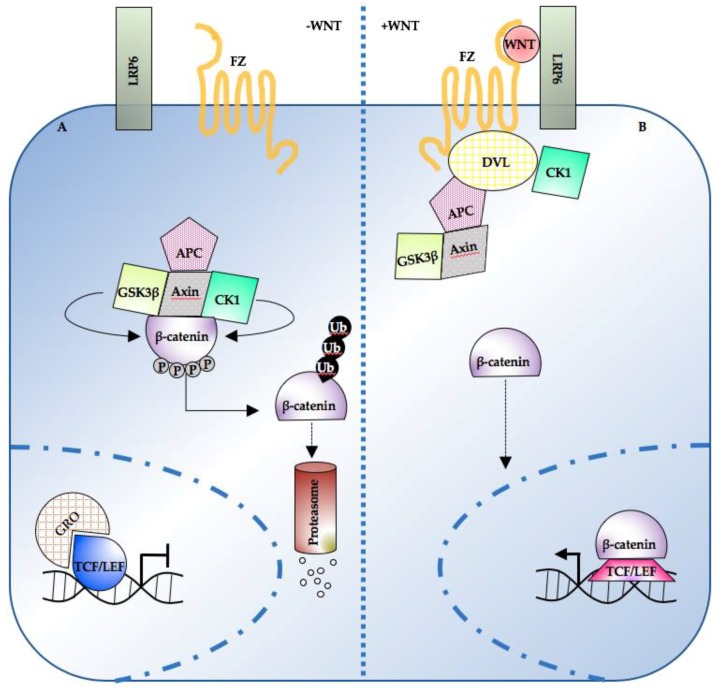
Overview of the Wnt/β-catenin pathway topology. (**A**) In the absence of WNT, cytosolic β-catenin is sequestered by the destruction complex and degraded following multiple rounds of phosphorylation and ubiquitination. Low nuclear β-catenin enables the TCF/LEF-mediated repression of target genes. (**B**) Following WNT ligand stimulation, the destruction complex is inhibited, and β-catenin accumulates. Nuclear β-catenin displaces the repressive complex from the DNA and drives target gene expression in co-operation with TCF/LEF transcription factors. TCF/LEF: T-cell transcription factor/lymphocyte enhancer factor; APC: adenomatous polyposis coli; CK1: casein kinase 1; DVL: dishevelled; FZ: frizzled; GSK3β: glycogen synthase kinase 3; LRP6: low-density lipoprotein receptor-related protein 6; Ub: ubiquitin; GRO: Groucho.

Meanwhile, nuclear TCF proteins associate with transcriptional repressors such as Groucho (Gro) and inhibit the expression of β-catenin target genes [56,57,58] (Figure 1A). When a Wnt ligand (such as Wnt-3a) binds to the seven-pass transmembrane receptor Frizzled (Fz) and its coreceptor, the low-density lipoprotein receptor-related protein 6 (LRP6), the signaling pathway is activated. Axin is recruited to the membrane and the PDZ-containing protein Dishevelled (DVL) is phosphorylated [59,60,61]. β-catenin is then released from the destruction complex; no longer exposed to kinase-mediated phosphorylation, it accumulates and can translocate to the nucleus. Therein, β-catenin has a dual function: it both displaces the transcriptional repressor complex from the DNA and, binding to TCF1 [62], LEF1 [38,43,63,64,65] and TCF4 [57,66,67], it activates the transcription of various target genes (Figure 1B). The latter, in turn, controls genes relevant for cell proliferation [68], stemness [69,70,71] and differentiation [72] (an updated list can be found online on the Wnt homepage, http://web.stanford.edu/group/nusselab/cgi-bin/wnt/).

The ability of the Wnt/β-catenin pathway to co-ordinate cell fate and homeostasis during development and in adult tissue has been extensively studied both in vitro and in vivo (reviewed in Reference [73]); furthermore, being the pathway misregulated in various diseases, including cancer and diabetes, various humanized antibodies and small molecules to antagonize its activity have been developed [37]. 

In this review, we focus on the specific dose- and dynamic-dependent Wnt/β-catenin signaling roles across systems and organisms.

## 2. Wnt/β-Catenin Pathway Levels, Dynamics, and Spatial Organization in Development

In vivo embryogenesis relies on the interplay of signaling cascades to activate tissue- and organism-specific differentiation programs. 

Multicellular-organism development relies on gene-expression patterns and on anterior–posterior (AP) polarity dictated by cellular positioning [74]. The Wnt pathway is one of the signaling pathways involved in the establishment of the AP axis and in supporting proper tissue development [75,76]. Mice lacking functional β-catenin protein are unable to undergo normal gastrulation [77]. Interestingly, excessive β-catenin accumulation is also associated with developmental defects, resulting in misregulated mesodermal differentiation [78]. These results suggest that the levels of β-catenin might need to be controlled and maintained within a certain threshold to properly support development. In this regard, Kemler and colleagues demonstrated that β-catenin levels vary during mouse development, and control all phases of embryogenesis [78,79]. Taking advantage of a conditional β-catenin allele and the ubiquitously active ROSA26 promoter, and generating different Cre lines, the authors showed a clear correlation between protein dosage and developmental progress in postgastrulation embryos, with different types of tissue requiring specific levels of β-catenin [79]. In mice, the Wnt/β-catenin pathway is fundamental for the proliferation of early pancreatic progenitor cells following cellular specification, and supports the undifferentiated state of pancreatic precursors; pancreas-selective β-catenin depletion results in a reduced number of pancreatic islets [80,81,82,83].

In *Xenopus*, low or high β-catenin accumulation levels in the anterior/posterior endoderm have been shown to maintain a foregut fate or to promote intestinal development, respectively [56,84]. In zebrafish, a key role for timing Wnt/β-catenin pathway activation was reported: upstream-pathway component APC was shown to rely on retinoic acid biosynthesis, and to regulate the transition from early gut endoderm into differentiated epithelial cells specifically postfertilization [85]. 

The importance of controlled β-catenin protein levels is also evident in murine and human hair-follicle homeostasis, where protein absence or overexpression results in inappropriate differentiation and/or tumor initiation [86,87]. Hair follicles rapidly form within the first two weeks after birth. Epithelial cells first massively proliferate and then differentiate, culminating into a final arrested phase known as telogen. In telogen, the epidermal bulge still retains stem cells that undergo several regenerative cycles during the lifespan [88,89]. In 1994, van Genderen and colleagues reported a detailed phenotypic analysis of the LEF1 transcription factor during development; using a targeted gene-inactivation approach, they demonstrated that different organs, which normally express LEF1 during development, were affected. The most severe phenotype was observed in the mammary gland, the teeth, whiskers, body hair, and the mesencephalic nucleus of the trigeminal nerve (TMN) [90]. Conversely, overexpression of LEF1 was shown to lead to de novo hair-follicle formation, similar to the overexpression of a constitutively stable β-catenin that induced uncontrolled ectopic hair growth and tumorigenesis [91]. Zonal activation of the canonical Wnt pathway was also found in the mesenchyme and epithelium of the developing tooth: using the Wnt/β-catenin pathway reporter Axin2-*lacZ,* both the epithelium and mesenchyme of the mandible, maxilla and nasal processes, but the frontonasal area showed Axin2 signal [92,93]. 

More recently, increased β-catenin expression was shown to force the differentiation of the embryonic ectoderm into hair follicles and promote de novo hair-follicle induction in adult skin; on the other hand, β-catenin depletion led to reduced proliferation of epithelial cells and premature catagen (i.e. regression phase prior to telogen) [94]. These observations indicate a temporal “wave” of β-catenin, with high/low levels in the initial/proliferative (and committed) phases, respectively [94]. To tip such balance between proliferation and differentiation [95,96], members of the Wnt family are dynamically expressed in developing hair follicles and skin, and the β-catenin protein itself shows dynamic changes in both accumulation levels and subcellular localization [97,98,99,100,101,102]. β-catenin knockdown experiments showed the canonical Wnt pathway is also important during hair-follicle regeneration; following intradermal injection of β-catenin siRNA into hair-depilated skin, hair growth was delayed of about 40 days [103]. 

### 2.1. Somitogenesis

Vertebrae formation starts from cellular precursors in a process known as the segmentation clock [104,105,106,107]; it is an oscillating network controlling the sequential subdivision of the vertebrate embryo elongating the body axis. During this process, somites are progressively formed from the anterior of the presomitic mesoderm (PSM), and elongate to form the body axis [108].

The mutual regulation of various signaling pathways and the resulting gradients and oscillations of molecules guide cell positioning and control somitogenesis [109].

Notch was the first signaling pathway shown to control the process, as the majority of the oscillatory genes are Notch-dependent [110,111,112,113,114,115,116,117,118]. Of note, Notch pathway impairment does not prevent segmentation [119], hinting the involvement of other pathways in somitogenesis. Herrmann’s group was the first reporting about the role of Wnt3a in the murine segmentation clock [119]. They discovered that Axin2, a negative regulator of the Wnt/β-catenin pathway [50,120,121] distributes over the PSM as a gradient and shows oscillatory dynamics in each cycle of somite formation. Axin2 periodic expression in the PSM could be to be due to its rapid and cyclic mRNA degradation, or to periodic production. Considering the topology of the Wnt/β-catenin pathway, the latter hypothesis is more plausible: being a transcriptional target of the canonical Wnt signaling, Axin2 is increased upon pathway activation and, in turns, can reduce pathway activation via its participation to the destruction complex, which reflects on decreased Axin2 transcription via a negative feedback loop [50,120]. Moreover, Axin2, similarly to Axin, might also be destabilized by Wnt signaling [122]. Crosstalk interactions with Notch signaling have been reported: the feedback inhibition of Wnt/β-catenin signaling via Axin2 can trigger Notch target gene activation [123]; thus, Wnt3a stimulation can activate Axin2 expression while inhibiting Notch signaling [119]. Fibroblast growth factor (FGF) signaling has also been observed in the PSM [124,125,126]: Sprouty2 or Dusp6 and Dusp4, all Fgf inhibitors, oscillate in phase with Notch cyclic genes due to further crosstalk interactions between the Notch and FGF pathways [126,127].

Recent in vivo studies from Wilson’s group reported differential levels of Wnt molecules during cell specification. Two subpopulations, both pluripotent, were identified in postimplantation epiblast stem cells (EpiSCs): a partially neuronal-like (Sox1+) fraction, expressing low Wnt/β-catenin levels, and a fraction of progenitor cells, with intermediate activation of the Wnt pathway. Further increase of Wnt/β-catenin signaling activity above a threshold irreversibly promotes mesendodermal and neuromesodermal differentiation [128]. 

### 2.2. Colon-Crypt Development and Homeostasis

The intestine has a peculiar functional architecture designed to maximize the available surface for absorbing nutrients and water. Epithelial cells invade the surrounding connective tissue to form tubular glands known as “crypts” [129], which are a reservoir of stem cells (intestinal stem cells, ISCs) supporting intestinal development and epithelium turnover [130,131]. The luminal portion of the mucosa is characterized by villi, fingerlike structures composed of terminally differentiated cells [132]. 

Intestinal-epithelium regeneration involves a series of events comparable to those taking place during intestinal development at the embryonic stage. ISCs migrate from the bottom of the crypt up to the villi; during this upward migratory process, cells are subjected to different stimuli blocking cell proliferation and promoting differentiation into all cell types required for intestinal functions (enterocytes, secretory goblet cells, Paneth cells, and endoenterocrine cells) [132,133]. In parallel, new crypts are generated through the fission process in order to support consecutive regenerative cycles [134].

The Wnt/β-catenin pathway has been demonstrated to control, in a dose-dependent manner, intestinal epithelium homeostasis in both health and disease. Dickkopf-1 (Dkk-1)-mediated Wnt pathway inhibition and Wnt pathway ablation are detrimental for crypt fission both in vitro and in vivo [135,136], and increased activation of the pathway (i.e., high β-catenin levels) and impaired cell-cell adhesion (i.e., high β-catenin levels and low E-cadherin) can trigger cancer formation [137,138]. In healthy conditions, the Wnt cascade tightly controls ISC overproliferation [139,140,141]: at the crypt base, nuclear β-catenin levels are higher, as well as in Paneth cells (positioned at the bottom of small intestinal crypts), while nuclear Wnt activity progressively decreases up the crypt–villus axis [142]. The resulting expression gradient and the different location of proliferative versus quiescent ISCs cause specific cell responses to Wnt signaling throughout the crypt [143]. Burgess and colleagues, using crypt explants and 3D confocal imaging, reported heterogeneous β-catenin and E-cadherin subcellular localization and patterning within the crypt, and proposed asymmetrical crypt budding in mice [144]; the group observed similar phenotypes in in vitro cultured 3D colonoids [145]. 

In the crypt, Wnt acts in coordination with other signaling pathways [146], namely: Notch [147] (regulating cell proliferation in the stem-cell niche [148]); Hedgehog (controlling proliferation of the ISC compartment and cell lineage differentiation in both small intestine and colon [149,150,151]); bone morphogenetic proteins (BMPs, belonging to the TGF-β cytokine family and required for intestinal cell-precursor proliferation, maturation, and terminal differentiation [152], and for the prevention of intestinal stem-cell overproliferation through inhibition of the Wnt gradient [146]); and Hippo/YAP (relevant for intestinal tissue regeneration [153]). The complex interaction between these pathways balances the progenitor number in the crypt, as well as their maturation and differentiation while moving up the crypt–villus axis, given cell position and associated signaling concentration gradients [146].

### 2.3. Central Nervous System

Wnt molecules and gradients regulate different aspects of nervous system development and function in vertebrates.

Exogenous overexpression of a stable (i.e., unresponsive to destruction complex-mediated degradation) form of β-catenin in hippocampal neuronal cultures causes an increased number of dendritic branches in a dose-dependent manner [154]. To test the requirement of endogenous β-catenin for proper dendrite development, Yu and Malenka impaired β-catenin binding to protein partners by overexpressing the intracellular domain of N-cadherin and found substantial reduction in the numbers of dendritic branches; this effect was shown to be independent of β-catenin transcriptional activity, as Lef1 overexpression did not cause a similar phenotype. The study also showed that cultures stimulated with potassium (which mimics the depolarizing effects of neuronal activity) present increased dendritic growth, partially through β-catenin signaling.

The canonical Wnt pathway also guides synaptogenesis [155,156,157]: WNT-7a promotes axonal remodeling by inhibiting the activity of the GSK3 kinase. Indeed, microtubule-associated proteins Tau, MAP-1B, and MAP-2 (microtubules stabilizer) are direct GSK3 substrates. Chemical inhibition of GSK3 recapitulates the phenotype of WNT-7a expression; in contrast, WNT-7a depletion results in reduced synaptic formation [158]. It was also found that, during synaptogenesis, N-cadherin/catenin complexes are initially uniformly distributed across all synaptic sites of neurons but are rapidly redistributed and restricted only to excitatory synaptic sites [159,160]. This process is also controlled by clusters of β-catenin and N-cadherin, which distribute in both pre- and postsynaptic compartments [160]. Additional studies indicated that dendritic morphogenesis depends on Wnt release from neighboring cells, and support the hypothesis of spatial diffusion of Wnt during brain development and activity [154,161,162]. 

The use of a β-catenin-activated promoter driving the expression of the β-galactosidase reporter helped in defying neurons that respond to activated β-catenin during mouse development. This study confirmed activation of the Wnt pathway in the mid-hindbrain and in the limb apical ectodermal ridge, and identified additional activated regions like the notochord and brain endothelia [75]. 

## 3. Wnt/β-Catenin Pathway Levels and Dynamics in Pluripotency, Differentiation, and Somatic-Cell Reprogramming

Embryonic stem cells (ESCs) are characterized by pluripotency (i.e., the potential to differentiate into any somatic cell type) and self-renewal (i.e., the ability of pluripotent cells to divide and maintain such potential). Pluripotency is a transient state in vivo; instead, ESCs can indefinitely be expanded in vitro, maintaining either ground state/naive or primed pluripotency states if isolated from the pre- or postimplantation epiblast, respectively [163]. Culture conditions are also crucial for ESC pluripotency maintenance. Focusing on the Wnt pathway, Sato and colleagues [164] first demonstrated that activation of the canonical pathway by GSK inhibition supports mouse ESC (mESC) in vitro pluripotency maintenance, even in the absence of the leukaemia inhibitory factor (LIF) in the culture medium. It was also demonstrated that Wnt pathway repression leads to mESC differentiation toward epiblast [165]. Such results prompted the establishment of protocols in which the canonical Wnt pathway is constantly activated chemically: the 2i/LIF culture medium combines LIF with two inhibitors (2i), PD0325901 and CHIR99021, repressing MAPK/ERK and GSK3β, respectively [166]. Importantly, 2i/LIF medium is serum free, and enables ground-state pluripotency maintenance, with overall homogeneous transcript levels of key pluripotency genes as compared to their pronounced heterogeneity and temporal fluctuations in FCS/LIF cultures [167,168]. In addition, 2i/LIF confers mESCs high efficiency in chimaera formation [169]. 

If and how β-catenin transcriptional activity is relevant for pluripotency is debated: in basal conditions (i.e., absence of pathway activators) it is negligible [170], and β-catenin establishes protein complexes with pluripotency master regulators Nanog and Oct4. Such results suggest that Wnt canonical pathway transcriptional signaling might be dispensable for mESC pluripotency [165,171,172,173,174,175]. Nevertheless, ground-state (e.g., 2i/LIF-cultured) mESCs show pronounced nuclear β-catenin accumulation and enhancement, but still heterogeneous transcriptional pathway activity [176]. It is still to be determined if there is a functional relationship between nuclear β-catenin accumulation levels and ground-state pluripotency. Instead, in FCS/LIF cultures, Kielman and colleagues demonstrated that β-catenin doses affect mESC capacity to differentiate into the three germ layers using APC mutants and teratoma-formation assays [177]. Notably, while promoting pluripotency maintenance in mESCs, Wnt/β-catenin signaling also drives the differentiation of primed cells (i.e., EpiSCs) toward the mesendoderm [72]. 

The canonical Wnt pathway is also relevant for somatic cell reprogramming (i.e., forced conversion of differentiated cells into pluripotent cells [178]). Wnt components are not directly targeted in the original cocktail of overexpressed transcription factors used by Yamanaka and colleagues to reprogram fibroblasts [179]. Nevertheless, is was shown that activation of the pathway can enhance the efficiency of both fusion- and factor-induced mediated reprogramming [180,181,182,183,184]. Interestingly, others and we reported a biphasic role of Wnt/β-catenin pathway in reprogramming, with its activation being beneficial only in the late stages of reprogramming [176,180,181]; this effect seems to not be related to β-catenin-mediated regulation of the cell cycle [68]. Furthermore, we reported detrimental effects of high levels of active β-catenin on cell-fusion-mediated reprogramming (i.e., reprogramming of mouse neural cells upon fusion with mESCs), which, instead, is enhanced by specific β-catenin doses [184]. 

Regarding human embryonic and induced pluripotent stem cells (hESCs and hiPSCs, respectively), recent studies indicate that their pluripotent state resembles that of mouse EpiSCs [185], as they retain some futures of primed pluripotency [186,187]. As compared to mESCs, hESCs and hiPSCs also respond differently to MEK–ERK pathway inhibition [188]; indeed, when they are cultured in 2i/LIF conditions, naive pluripotency is not supported [189]. Nevertheless, all culture protocols recently proposed to maintain hESCs and hiPSCs in the naïve state of pluripotency rely on Gsk3 inhibition [73]; however, it was also reported that the canonical Wnt pathway promotes hESC differentiation [190]. Ectopic expression of OCT4/KLF4 or KLF2/KLF4 or KLF2/NANOG in human cells can stabilize their pluripotency when cultured in 2i/LIF [191] and 2i/LIF/aPKCi [192]. Recently, a transgene-independent naïve-state medium was proposed: the so-called naive human stem-cell medium (NHSM) contains 2i/LIF further supplemented with p38, Jun N-terminal kinase (JNK), aPKC and RHO-associated protein kinase inhibitors, and a low amount of FGF2 and Activin A or TGFβ1 [188]. However, this culture condition causes loss of DNA imprinting [193]; further studies are needed to define alternative culture protocols that can promote and support naïve pluripotency without erasing germline memory.

WNTs and their effectors are also crucial for potency maintenance in adult stem cells [165,194,195,196,197]. In vivo analysis of various APC mutants suggested that specific signaling levels are associated with cell specification into hematopoietic stem cells, myeloid progenitors, and early thymocytes during haematopoiesis [198]. Bone marrow (BM) from the Axin2^LacZ^ Wnt reporter mouse model showed differential sensitivity in reporter activity when stimulated with the canonical Wnt pathway. Furthermore, when studying hematopoietic stem cell (HSC) differentiation in the presence of varying Wnt/β-catenin activity, mild/intermediate levels were shown to enhance clonogenicity and myeloid differentiation, while high levels strongly reduced the number of colonies; only a mild increase was able to confer increased HSC repopulation potential [198]. 

## 4. β-Catenin and Cancer 

Homeostasis tightly controls the number of cells within tissue, balancing cell growth and survival. Genetic mutations or sporadic events can result in uncontrolled cell proliferation and/or increased cell survival, which can ultimately lead to cancer initiation [199,200]. Often, dysplastic events involve the stem-cell compartment of the tissue, which is more susceptible to mutagenic events. β-catenin is rarely mutated in cancer, but mutations of its main protein partners and gaining function effects can confer enhanced stability to β-catenin, causing its aberrant accumulation [201,202].

Colorectal cancer (CRC) is the first and most characterized cancer model involving the canonical Wnt pathway. Because of APC mutations, β-catenin levels increase, and pro-proliferative genes are activated following its nuclear translocation [38,202]. A similar cancerogenic phenotype is also induced by mutations in others protein members of the canonical Wnt pathway, such as human naked-cuticle homolog NKD1 [203] and protein phosphate 2A (PP2A) [204,205]. Mutations of NKD1 have been frequently found in CRC: loss-of-function studies, using different mutants of the wild-type gene, showed defective inhibition of Wnt/Dvl signaling [203]. NKD1 is involved in Dvl proteasomal-mediated degradation; therefore, the inability to destabilize Dvl results in aberrant β-catenin accumulation and increased cell proliferation. PP2A functions are related to the control of many signaling cascades by opposing the activity of protein kinases (as reviewed in [206]). PP2A binds to different components of the Wnt pathway, including APC and Axin, and affects its activity both upstream and downstream of β-catenin [207,208,209]. Additionally, PP2A has binding domains for NKD1 [210] and coordinates β-catenin/E-Cadherin binding with a direct effect on the epithelial–mesenchymal transition happening during cancer initiation, ultimately balancing the ratio between complexed and free β-catenin [211]. Indeed, mutants of the PP2A subunit Calpha fail mesoderm differentiation and are lethal [211]. β-catenin accumulation has also been associated with non-CRCs of the gastrointestinal tract, including the liver and the biliary tract [212,213,214], the connective tissue [215], glial cells [216], and the hematopoietic system [217]. 

A study on a cohort of human gastric adenocarcinomas revealed β-catenin nuclear accumulation in 29% of samples, whereas 71% only showed membrane β-catenin staining; this study did not investigate a possible correlation between β-catenin levels and/or intracellular localization and cancer severity [212]. Similar results were also found in patients with hepatocarcinoma (HCC), where 19% of samples showed β-catenin presence in the nucleus [213]. Desmoid-type fibromatosis showed β-catenin nuclear accumulation in approximately 50% of a tumor, and this observation was consistent across patients with wild-type β-catenin (where accumulation is likely caused by mutations in Wnt partners) or mutated β-catenin [215]. Finally, β-catenin was found to be the driving force of mixed-lineage leukaemia (MLL) stem-cell (LSC) development. So’s research group characterized β-catenin translocation and canonical Wnt pathway activation during the pre-LSCs to LSCs transition (i.e., the process that originates aggressive and drug-resistant leukaemia) and demonstrated that the Wnt pathway is highly activated when tumorigenesis starts, while β-catenin knockdown impairs both murine and human MLL cell proliferation [217]. 

The role of β-catenin in cancer is not only restricted to the proliferative advantage of cancer cells, but also to their ability to colonize surrounding tissue, potentiating metastasis formation [218,219,220] and immune-system evasion [221,222,223,224]. Transcriptional profiling of metastatic vs. nonmetastatic breast-cancer cells showed overexpression of the canonical Wnt pathway (i.e., β-catenin and LEF1), β-catenin target genes (i.e., c-Myc and cyclin D1), and Wnt ligands (i.e., Wnt3a-7a); secretion of Wnt ligands might be responsible for the activation of the Wnt pathway in cells distant from a primary tumor [218]. An alternative study about the metastatic power of melanoma cells showed that, when overexpressed into mice, β-catenin acts in two phases: it initially reduces cell migration and only in the second stage it promotes metastatic spread [219]. Reduced cell migration can be explained by an autonomous property of cancer cells by which they need to be less motile in order to colonize the primary tissue before spreading to others [219]. These observations can be reconsidered in view of recent findings of β-catenin-mediated immune-system elusion of cancer cells: melanoma specifically expressing constitutive active β-catenin does not present any T-cell infiltration [223]. Manicassamy and colleagues recently proposed a molecular mechanism for tumor-induced immunosuppression, in which increased β-catenin activity in tumor-resident dendritic cells (DCs) can lead to enhanced activity of vitamin A-metabolizing enzymes. This results in faster retinoic acid (RA) metabolism, with RA driving regulatory T-cell responses and immune tolerance [225]. Interestingly, in the same work, β-catenin inhibition was shown to reduce tumor growth, opening new therapeutic avenues for combined targeting of Wnt/β-catenin and RA metabolism pathways.

Although β-catenin is involved in many phases of cancer progression and higher levels worsen prognosis [220,226], little is known on whether different amounts of Wnt protein accumulation relate to specific phases of carcinogenesis. 

## 5. Conclusions and Future Directions

**Figure 2:**
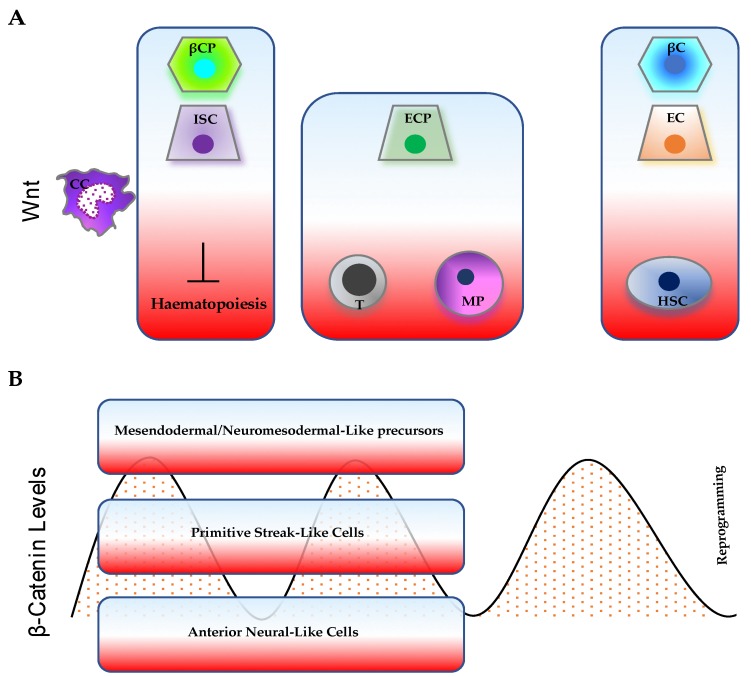
Representative cellular processes influenced by (**A**) Wnt gradient and (**B**) time-varying β-catenin levels. The cellular response can depend on both the levels of Wnt/β-catenin pathway activity and on cellular/tissue context. (**A)** High/intermediate Wnt levels support both intestinal stem-cell (ISC) and β-cell progenitor (βCP) expansion; low Wnt levels stimulate terminal differentiation of enterocytes (EC) and β cells (βC), but sustain hematopoietic stem-cell (HSC) maintenance. Intermediate Wnt levels are mostly associated with blood-cell commitment (T cells, T; myeloid progenitors, MP) and enterocyte-progenitor (ECP) differentiation. (**B**) β-catenin oscillations control embryo patterning. High or low levels of β-catenin can either promote or impair somatic cell reprogramming, respectively.

The pleiotropic roles of the Wnt/β-catenin pathway in regulating multiple processes, including embryogenesis, pluripotency, differentiation, and cancer, have been reviewed. It appears clear that the functions of the pathway are highly cell- and context-dependent [227]; across different systems, dose- and dynamic-dependent functions have also been shown (Figure 2).

Feedback loops in pathway topology [120,228] and crosstalk interactions with other pathways across species and systems [125,229,230,231,232,233] might be the cause of switch and oscillatory-like pathway behaviors. Such nonlinear dynamics could explain β-catenin’s dual role in systems for which its heterogeneous levels have been reported but not functionally characterized, such as mESCs cultured in both naïve (i.e., serum-based) and ground-state (i.e., serum free) pluripotency media [176,234].

Mathematical models can be instrumental in providing quantitative insights into the characteristics and dynamics of signaling pathways and coupled dynamic processes, allowing to test hypotheses and generate in silico predictions [235]. While the validity of modeling results inevitably depends on model parameters, assumptions, and structure [236], computational representations of cells can be instrumental when combined with ad hoc experimental validations. An elegant work confirmed experimentally in mammalian cells predictions of a computational model (developed using *Xenopus* embryos data [237]) that the canonical Wnt transcriptional system can respond to β-catenin fold changes instead of its absolute levels, possibly due to incoherent feed-forward loops in pathway topology [238]. The canonical Wnt pathway is coupled to the cell cycle (see References [239,240] for a review). Despite its established promitotic role, recent studies [102] have shown it can also promote expression of cell-cycle negative regulators in mESCs. Given this, and the aforementioned role of pathway gradients in tissue organogenesis and homeostasis, as well as in cell-cell adhesion, multiscale computational models and agent-based cell-simulation frameworks might be needed to better formalize signaling-pathway dynamics, thanks to their ability to describe cell mechanics, 2D/3D tissue geometries, single-cell gene expression and its coupling to cell proliferation [241,242,243].

Novel experimental techniques can also be instrumental to developing a better and quantitative understanding of the role of spatial and temporal Wnt/β-catenin (and other signaling) pathway dynamics in controlling tissue homeostasis. Three-dimensional in vitro cell clusters and organoids enable studying processes like organogenesis and cancer development in a fully controllable setting [244], especially when combined with genome-modification strategies such as viral transgene delivery and CRISPR/Cas9 technology. Successful examples include the development of bladder cancer-cell-derived organoid cultures to study the link between Wnt/β-catenin pathway activation and cellular organization and proliferation [245]; analysis of precardiac spheroids to study Wnt and BMP role in the specification of two-cardiac origin [246]; and the use of intestinal organoids to study epithelial self-organization in the gut [244,247,248].

Live-cell imaging, combined with precise perturbations of signaling-pathway dynamics, might contribute significantly to dissect how the latter control cell fate. In a recent work, a microfluidics/imaging-based approach was developed to entrain oscillations of Notch and Wnt signaling to predetermined external periodic forces in the anterior monolayer PSM [123]; this experimental setup allowed to quantitatively address important questions about the crosstalk between the two pathways, and the role of relative timing between individual pathway oscillations in the control of mesoderm segmentation.

The use of feedback control, recently combined with microfluidics/imaging platforms to precisely regulate gene expression in living cells [249], and with synthetic biology tools to recreate, in a fully controllable and reproducible way, signaling-pathway activity [250,251], might open important avenues to quantitatively study dose- and dynamic-dependent signaling functions across biological systems, and develop ad hoc interventions in case of malfunction.
